# Families’ Perspectives on Physical Restraint Use in Older Adults: A Scoping Review

**DOI:** 10.1093/geroni/igaf122.3589

**Published:** 2025-12-31

**Authors:** Eun-Hi Kong

**Affiliations:** Gachon University, Incheon, Republic of Korea

## Abstract

Despite many harmful effects, physical restraints have been widely used for older adults in many care settings. Most earlier studies have focused on formal caregivers and little is known about family caregivers of older adults with physical restraints. Therefore, the aim of this review to synthesize research evidence of the literature related to families’ perspectives on physical restraint use. Systematic literature search was conducted using electronic database search including MEDLINE, EMBASE, CINAHL, Cochrane Library, KoreaMed, RISS, and Google scholar from the earliest year to July, 2025. The combination of the keywords related to physical restraint, family, and older adults was used. Using EndNote21, search results were imported and duplicates were removed. Grey literature as well as published articles were included in this review. This review followed the JBI guidance and the PRISMA-ScR extension for scoping review. Eleven studies were included in this review. The studies were conducted in many countries on three continents. Six studies were quantitative studies (non-experimental studies) and five studies were qualitative descriptive studies. Family caregivers of older adults receiving home care or long-term care were included. This review found that many family caregivers had mixed perceptions towards physical restraint use on their loved ones. Also, this review pointed out family caregiver’s lack of education regarding physical restraint. This review shows that there is a dearth of intervention studies (experimental studies) targeting family caregivers for physical restraint reduction. Future studies are required to develop and evaluate educational programs of restraint-free care and person-centered care for informal caregivers.

